# Detection of Thermal Sublethal Injury in *Escherichia coli* via the Selective Medium Plating Technique: Mechanisms and Improvements

**DOI:** 10.3389/fmicb.2016.01376

**Published:** 2016-08-30

**Authors:** Laura Espina, Diego García-Gonzalo, Rafael Pagán

**Affiliations:** Departamento de Producción Animal y Ciencia de los Alimentos, Facultad de Veterinaria, Instituto Agroalimentario de Aragón – IA2, CITA-Universidad de ZaragozaZaragoza, Spain

**Keywords:** sublethal injury, osmoregulation, selective media, *Escherichia coli*, flow cytometry

## Abstract

In food preservation, the synergistic combination of different technologies aims to maximize the total lethality of the process and minimize the intensity of each hurdle. This is especially the case when at least one of the treatments can cause sublethal (reparable) injury in a great proportion of the population, so that sublethally injured cells can end up being entirely inactivated by the other hurdle(s). The selective medium plating technique (SMPT) is extensively used to enumerate bacterial sublethal injury after inimical treatments, being sodium chloride added to the recovery medium to detect damaged bacterial envelopes. However, little work has been done to explain the reasons for the inability of sublethally injured cells to outgrow in selective agar media, whereas they are able to grow in non-selective agar. In the present paper, the performance of SMPT on *Escherichia coli* cells after heat treatments is explored by applying different selective agents in the recovery media, using mutants lacking factors involved in osmoregulation, and also by examining the integrity of the cytoplasmic membrane. In view of the results, the possibility of a specific toxic effect of Na^+^ as the main mechanism under SMPT was discarded, since the same level of sublethal injury was detected using KCl instead of NaCl. The synthesis of the osmoprotectant trehalose determined the maximum osmotolerance of intact cells to the selective agents, but was not crucial in the quantification of sublethal injury. Moreover, for the first time, the extent of sublethal injury detected via SMPT was directly correlated with the physical loss of integrity of the cell membrane in 99.999% of the initial population. This was achieved through statistical analysis of flow cytometry data using propidium iodide-exclusion technique when that dye was added before thermal treatments. The present work confirms the adequacy of SMPT as a tool for detecting the occurrence and quantity of sublethally injured cells after thermal treatments and thus, for efficiently designing the combination of heat with other preservation techniques. We also propose the study of statistical analysis from flow cytometry data for a more rapid quantification of bacterial sublethal injury in a broad detection range.

## Introduction

In bacteriology, viability has been traditionally defined and measured as the ability of organisms to self-replicate in culture media (Bogosian and Bourneuf, 2001; Nyström, 2001). However, it has long been known that the failure of a bacterial cell to produce a colony on a standard nutrient plate may not necessarily mean that the cell was dead at the time of sampling (Nyström, 2001). For instance, microorganisms that are metabolically active despite their inability to grow in laboratory culture media are said to be in a “viable but non-culturable” (VBNC) state, which, under harsh environmental conditions, can be triggered as a survival mechanism (Bogosian and Bourneuf, 2001). On other occasions, exposure to chemical or physical processes can lead to the sublethal injury of bacterial cells: this state is considered to be transient, since cells are able to repair their damages and resume growth if suitable environmental conditions emerge (Mackey, 2000).

In food preservation it has been demonstrated that, once one has applied preservation treatments to control bacterial food contamination, a considerable proportion of the population may become sublethally injured in addition to both the surviving (non-injured) and the inactivated populations (Wesche et al., 2009). The adequate identification and quantification of the sublethally injured population plays a key role in food safety. Since damaged cells are not generally able to grow on the conventional selective enrichment media used in the food industry (Restaino et al., 2001), they can remain undetected, subsequently repair their damages and reach infective concentrations (Mackey, 2000). On the other hand, according to the “hurdle effect” (Leistner and Gorris, 1995), repair of sublethally injured cells after a preservation treatment can be adequately prevented by the combination of additional preservation agents (hurdles) that interfere with cellular homeostasis maintenance, thereby synergistically increasing the combined process’s global lethality (Mackey, 2000).

In this regard, although new methods are being developed for the detection of sublethally injured bacteria (Back et al., 2012; Gelaw et al., 2014), the most widely used strategy among microbiologists is still differential enumeration on non-selective and selective agar, following the so-called selective medium plating technique (SMPT; Mackey, 2000). For this purpose, out of all possible selective agents, subinhibitory concentrations of sodium chloride have been consistently incorporated in the recovery medium (Chilton et al., 2001; Ulmer et al., 2002; Miller et al., 2006). It is believed that the increase in osmotic pressure caused by the addition of sodium chloride explains the selective outgrowth of only those cells whose cytoplasmic membrane remains intact (Mackey, 2000).

Despite the observed selective effect of the osmolyte NaCl on bacterial growth, little research has been done to study the osmoregulatory mechanisms of sublethally injured cells and, therefore, to find out more about their ability to maintain selective permeability after different stresses. In intact bacteria, an osmotic upshock unleashes a cascade of events intended to maintain turgor pressure within limits by regulating the total osmotic solute pool in the cytoplasm (and in the periplasm in Gram-negative bacteria; Wood, 2011). As the osmolality of the surrounding environment increases, turgor pressure drops and growth slows or halts (Wood, 2011). The most rapid response to this osmotic upshock is an increase in potassium ion influx that increases cytosolic osmolality (Wood, 2011). Since high intracellular concentrations of K^+^ interfere with many important cellular functions, the cell starts to accumulate large quantities of so-called compatible solutes, which are more congruous with its physiology (Wood et al., 2001). The compatible solute trehalose is synthesized (via Ots system) and accumulated up to levels that may comprise as much as 20% of cytoplasmic osmolality under conditions of high osmolality (Wood, 1999). Other compounds, when present externally (such as glycine betaine), can be incorporated via transporters such as BetT or ProP (Lucht and Bremer, 1994; Wood, 2011), leading to a decrease in trehalose levels and stimulating bacterial growth rates under hyperosmotic conditions. It has been estimated that after 1 h of osmotic stress, a cell’s physiology and structure are largely restored via these osmoregulatory systems (Wood, 1999). A better knowledge of the interaction between cellular osmoregulatory mechanisms and the permeabilization of the cytoplasmic membrane and how they influence bacterial ability to outgrow in selective media could facilitate the estimation of sublethal injury and, therefore, help us improve the design of food preservation processes.

In the present study, SMPT is applied as the primary technique to detect and quantify the proportion of sublethally injured cells in their cytoplasmic membrane after exposure to a lethal stress. Thermal treatment was selected as the lethal stress, since it is the most studied and best understood treatment known to sublethally injure microorganisms (Wesche et al., 2009). It should be noted that mild thermal treatments applied in fluid environments have been demonstrated to disturb the permeability of the outer membrane earlier and more intensely than the permeability of the cytoplasmic membrane (Mackey, 2000; Shigapova, 2004); thus, the outer membrane does not interfere with the detection of sublethal injury in the cytoplasmic membrane. The microorganism *Escherichia coli* was also selected, since it is the model microorganism for studying bacterial osmoregulation (Shabala et al., 2009). Besides, the availability of a great variety of *E. coli* mutants lacking factors involved in the osmoregulatory system (Baba et al., 2006) can be used to determine those factors’ role in SMPT.

The primary objective of this study was (i) to gain a better understanding of the mechanisms underlying SMPT by trying to identify which bacterial osmoregulatory mechanisms or physical structures are modified by heat and are thus responsible for the prevention of bacterial growth in selective media. Additionally, we aimed (ii) to improve traditional SMPT by testing the effect of different variations in the composition of the recovery media, and also (iii) to explore the possible use of flow cytometry as a complementary technique to assess sublethal injury.

## Materials and Methods

### Preparation of Media

Minimal medium M9 was chosen as the broth and treatment medium, since it is commonly used for the culture of *E. coli* (Neidhardt et al., 1974), and because its minimal composition reduces the presence of osmolytes or osmoprotectants influencing the osmoregulation processes. M9 minimal broth was prepared following the steps indicated in Maniatis et al. (1982): its composition is of 38 mM Na_2_HPO_4_, 20 mM KH_2_PO_4_, 7.7 mM NaCl, 17 mM NH_4_Cl, 1 mM MgSO_4_, 0.1 mM CaCl_2_, and 0.2% glucose.

Regarding the recovery media, both minimal and rich agar plates were prepared to cover a whole range of culture conditions, as both types are commonly used in the study of sublethal injury (Wesche et al., 2009). In addition to the ingredients in M9 minimal broth, the M9 minimal agar medium contained 15 g/L of Agar Technical No. 3 (Oxoid, Basingstoke, UK).

Tryptic soy agar (Biolife, Milan, Italy) plus 0.6% of yeast extract (Biolife; TSAYE) was selected as the rich recovery medium, given its widespread use in the enumeration of bacterial injury (Miller et al., 2006; Noriega et al., 2013). Preliminary experiments showed that recovery in M9 minimal agar medium after different thermal treatments yielded similar counts than in TSAYE (data not shown).

Although, NaCl is the solute most commonly used to inhibit growth in selective agar media when evaluating sublethal injury in the cytoplasmic membrane, we also tested the osmolytes KCl and saccharose. With the objective of determining the influence of the type of osmolyte in the detection of sublethal injury, each solute was added in the concentration required to achieve the same osmolality values in the agar medium. For this purpose, the osmolality values of the agar (Os/kg of M9 agar medium) were chosen to correspond with those created by the addition of 1–6% of NaCl, and resulted in a range of 0.34–2.05 Os/kg of agar medium. The KCl and saccharose concentrations required to achieve such osmolality values were 1.27–7.68% KCl or 11.63–70.17% saccharose.

Betaine was added as osmoprotectant at 1 mM, following the lines of previous research (Le Rudulier et al., 1984; McLaggan et al., 2002). Higher concentrations were not proven more effective to osmotically protect cells (data not shown).

### Micro-Organisms and Growth Conditions

The strains used were *E. coli* BW25113 and its deleterious mutants *E. coli* Δ*otsA*, Δ*proP*, Δ*nhaA*, Δ*nhaB*, and Δ*nhaR*. While factors OtsA and ProP are involved in the synthesis of trehalose and the uptake of betaine respectively, the different subunits of the factor Nha are involved in the excretion of Na^+^. All strains were obtained from the KEIO collection (Baba et al., 2006).

The cultures were maintained in cryovials at -80°C prior to use. Broth subcultures were prepared by inoculating one single colony from a plate in a 50-mL flask containing 10 mL of sterile M9 minimal medium. After inoculation, the flasks were incubated overnight at 37°C. With these subcultures, 250-mL Erlenmeyer flasks containing 50 mL of M9 medium were inoculated into a final concentration of 3 × 10^6^ CFU/mL. These flasks were incubated with agitation (130 rpm; Selecta, mod. Rotabit, Barcelona, Spain) at 37°C until stationary growth phase was reached (24 h).

### Thermal Treatments

Before inoculation, cultures were centrifuged at 6000 × *g* for 5 min and resuspended in the treatment medium (M9 medium).

For the preparation of heat-treated samples, 0.1 mL of culture at 10^9^ CFU/mL was added to a tube containing 0.9 mL of M9 medium tempered at 55 ± 0.2°C or at 53, 57, or 59 ± 0.2°C (FX Incubator, A. F. Ingeniería S. L., Valencia, Spain). The actual temperature was controlled with a thermocouple wire introduced in a 0.9 mL M9 broth test tube inside the incubator. After each individual treatment interval, samples were taken, immediately placed on ice, and adequately diluted in 0.1% w/v peptone water (Biolife). Survivors were evaluated as explained below.

Exceptionally for an experiment aimed to compare inactivation kinetics in the absence and presence of the osmoprotectant betaine, survival curves to heat treatments were obtained in a specially designed thermo-resistometer, as previously described (Condón et al., 1993). This device has a thermocouple (Pt 100) to monitor the temperature during heat treatment and for the injection of inoculum. Once the temperature had stabilized (at 58, 61, 64, 67, or 70°C), 0.2 mL of culture was injected via a solenoid-valve-operated automatic syringe into the 400-mL treatment chamber containing the treatment medium under constant agitation. Samples were taken at regular intervals and survivors were evaluated as explained below.

### Collection of Samples, Counts of Culturable Cells and Quantification of Sublethally Injured Cells

In order to quantify bacterial cell injury, in a first step the maximum non-inhibitory concentration (MNIC) of each osmolyte in M9 agar medium was determined. To achieve this, untreated cells were spread plated onto M9 agar media with different concentrations of each solute (NaCl, KCl, or saccharose), and plates were incubated at 37°C for 48 h. According to previous work (Cebrián et al., 2014), the MNIC was defined as the highest concentration which inhibited less than 20% of the initial untreated bacterial population.

After treatments, 0.02 mL volumes of adequately diluted samples (using M9 broth as the dilution medium) were spread on the surface of prepared M9 agar and/or TSA plates, in both non-selective and selective plates. Exceptionally, samples treated with the thermo-resistometer were poured either directly on plates (for treatment temperatures of 58, 61, and 64°C) or were pour-plated after having been collected in agar-medium-containing tubes placed on a rotating carousel (for experiments performed at 67 and 70°C). This sample-collection device allowed for the characterization of survival curves despite the high inactivation rates at these treatment temperatures.

In all cases, plates were incubated at 37°C for 48 h. Previous experiments showed that longer incubation times did not influence the amount of surviving cells regardless of the added osmolyte. For each dilution, 10–200 colonies were counted on the surface of the agar medium in spread-plated samples. For pour-plated samples, colonies were counted with an improved Image Analyzer Automatic Counter (Protos; Analytical Measuring Systems, Cambridge, UK) as described in earlier work (Condón et al., 1993). Taking into account the initial cell concentration in the thermoresistance experiments (10^8^ CFU/mL), the detection limit was of 5 log_10_ cycles.

Inactivation was expressed in terms of the extent of reduction in log_10_ counts (CFU) after any treatment. Survival curves were obtained by plotting the decimal log_10_ fraction of survivors versus the treatment time for each independent experiment. The extent of sublethal injury was expressed as the difference between the log_10_ count (CFU) on non-selective medium (M9) and the log_10_ count on selective media. Likewise, the percentage of injured cells at each treatment time corresponded to the following equation (Busch and Donnelly, 1992):

(1) $$\begin{array}{c} \%\mathrm{Injured}\mathrm{cells}=\;1\;-\;\left(\frac{CFU/mL_{selective}}{CFU/mL_{nonselective}}\;\times \;100\right) \end{array}$$

According to this representation, “2 log_10_ cycles of injured cells” means a 2-log_10_ difference in the count on selective and non-selective media, or that 99% of survivors were sublethally injured.

Experimental data were obtained from at least three independent experiments performed on different days.

### Thermotolerance Parameters

When appropriate, survival curves were fitted by a model based on a Weibull-like distribution, which was chosen based on their linear and concave upward profiles. For this investigation we used the equation proposed by Mafart et al. (2002) (Eq. 1):

(2) $$\begin{array}{c} Log_{10}\frac{N_{t}}{N_{0}}\;=\;-{\left(\frac{t}{\delta }\right)}^{p} \end{array}$$

where t is the treatment time (min); *N_t_* and *N_0_* are the population densities (CFU/mL) at time t and time 0, respectively; and δ and ρ are two characteristic parameters of the equation. The δ value is called the time to the first decimal reduction (time necessary to inactivate the first 1 log_10_ CFU of the microbial population). The ρ value is the shape parameter.

### Determination of the State of Cells Grown in Agar Media Containing NaCl

To determine the state of cells (viable, inhibited, or inactivated) when grown in agar media with different concentrations of NaCl, wild type (WT) or Δ*proP* untreated or heat-treated cells (10 min at 55°C) were carefully sampled onto plates with M9 agar medium added with 0–10% NaCl. The initial sampled concentration of cells was 5 × 10^6^ CFU/plate, and plates were incubated for 48 h. After that first incubation, a method was developed to recover colonies from colony-lacking plates in a highly reproducible way. For this, 4 g of agar of each plate from the first incubation were carefully extracted, placed in sterile plastic bags with peptone water 0.1%, and homogenized for 20 s at 230 rpm in a stomacher laboratory blender (model 400, Tekmar, Co., Cincinnati, OH, USA). Next, 1 mL-aliquots were spread plated onto non-selective M9 agar plates and incubated once more for 48 h. After that second incubation, the surface of the plates was visually inspected and classified into positive growth (presenting a high enumerable concentration of CFU/plate) or negative growth (with less than 5 CFU/plate).

For each degree of NaCl concentration in the first agar medium, the state of intact or heat-treated cells was classified as viable (when colonies were observed after the first incubation at the expected concentration of 4 × 10^6^–5 × 10^6^ CFU/plate), inhibited (colony-lacking plates after the first incubation but with positive growth after the second incubation) or inactivated (colony-lacking plates after the first incubation and with negative growth after the second incubation). Data shown are results from a representative experiment repeated twice with similar results.

### Measurement of Cell Permeabilization via Propidium Iodide (PI) Uptake

For the evaluation of cell permeabilization, PI at a concentration of 0.08 mM (Pagán and Mackey, 2000) was added to the treatment medium prior to the thermal treatment. Alternatively, PI was not added before treatments and was incorporated immediately after each treatment in order to obtain additional information. Cell permeabilization was analyzed by fluorescence microscopy and by flow cytometry.

For the analysis under the fluorescence microscope, treatments were applied at 55°C for 0–5 min. For the flow cytometry analysis, the treatment temperature was 53°C in order to achieve longer intervals between samples. After each treatment, samples were immediately placed on ice, subsequently incubated for 15 min at 20°C, centrifuged at 6000 × *g* for 5 min, and washed three times. For the flow cytometry analysis, samples were also immediately fixated with a preparation of 4% paraformaldehyde in PBS, washed three times and diluted to a concentration of 10^5^ CFU/mL in PBS.

The measurement of cell permeabilization with the fluorescence microscope (Nikon, Mod. L-Kc, Nippon Kogaku KK, Japan) was performed by direct counting of non-fluorescent and fluorescent bacteria at 1000× magnification. About 200 bacteria were visible in a field of vision, and bacteria from five fields of vision were counted per sample and replicate.

For each sample analyzed by flow cytometry, 10,000 events were counted using a MACSQuant Analyzer (Miltenyi Biotec, Cologne, Germany) flow cytometer. Fluorescence data were collected using the 488 nm excitation laser and the 614–650 nm filter, corresponding to the B2 channel in the MACSQuant Analyzer.

The evaluation of PI uptake by each of those two techniques was run in triplicate on separate days.

### Statistical Analyses and Management of Flow Cytometry Data

For kinetics analysis of the data from survival curves, the least-squares criterion of the GraphPad PRISM program (GraphPad Software, San Diego, CA, USA) was used. This program was also used to perform ANOVA and *t*-test; differences were considered significant if *p* ≤ 0.05.

Data from flow cytometry was analyzed with FCS Express 5 (De Novo Software, Los Angeles, CA, USA). For the measurement of fluorescence intensity, the parameter “area under the curve” was chosen over “pulse height” in order to consider not only the maximum fluorescence of each event, but also the time required to collect data. No gates were created to obtain histograms or statistical data thereof. Before running the actual samples, unstained and stained cells were analyzed in the flow cytometer to establish the adequate threshold levels for the identification of “events” as “cells” and for the sensitivity of the fluorescence signals.

## Results and Discussion

The SMPT allows for the estimation of the occurrence of sublethal injury after each treatment by measuring the difference between the inactivation level achieved in a selective medium and the inactivation level achieved in a non-selective medium (Mackey, 2000). In order to assess the damage in the cytoplasmic membrane, sodium chloride is added at its MNIC, so that only non-damaged cells are able to multiply.

**FIGURE 1:**
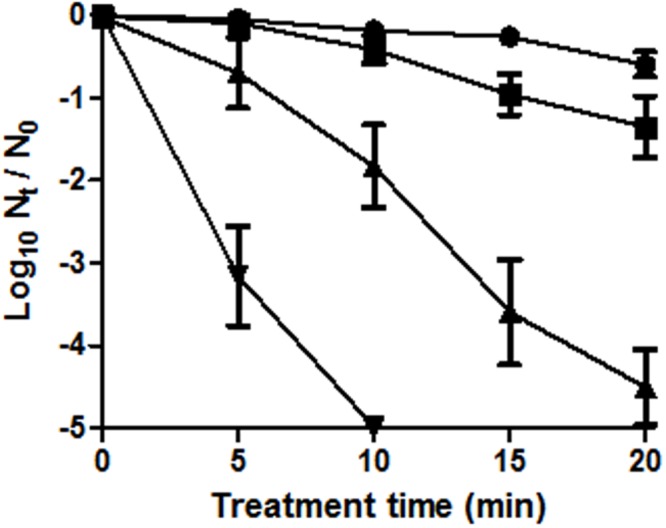
**Survival curves of *Escherichia coli* BW25113 (initial concentration: 10^8^ CFU/mL) to a heat treatment at 55°C in M9 broth for different treatment times.** *E. coli* cells were cultured in M9 agar (non-selective agar) (●) or M9 agar supplemented with 1 (■), 2 (▲), or 3% (▼) NaCl. Error bars represent standard deviation of the mean from three replicates.

In the present study we primarily intended to offer a simple example of the performance of SMPT after thermal treatments on *E. coli*. Cells were recovered in M9 agar with 1, 2, o 3% NaCl. Concentrations over 3% NaCl (MNIC) in the agar inhibited the growth of untreated cells. The results, depicted in **Figure 1**, show that after 10 min of treatment less than 0.2 log_10_ cycles of the initial population failed to grow in non-selective agar medium. However, when recovered in agar medium containing 1, 2, or 3% NaCl, the population of cells unable to grow increased in 0.2, 1.6, or 4.8 log_10_ cycles respectively. This graph demonstrates that even a very mild thermal treatment can result in an increased sensitivity to NaCl in the agar media in the majority of the initial bacterial population, corresponding to sublethally injured cells.

On the other hand, there was a gradual inverse relationship between the osmolality of the recovery medium and the proportion of growing cells. Therefore, the more severely injured cells are, the lower the NaCl concentration required to prevent their growth – which fits perfectly with the previously stated hypothesis of the coexistence of different levels of damage, from minor to eventually lethal (Wesche et al., 2009; Noriega et al., 2013).

### Insights into the Failure of Sublethally Injured Cells to Grow on Osmotically Selective Media

The increased sensitivity of cells to NaCl after thermal treatments does not have a clear origin, although it has been traditionally ascribed to the loss of permeability control, leading to their irreversible inactivation (Mackey, 2000). However, little research has been done to identify mechanisms or structures that are damaged by heat and therefore prevent bacterial growth in the presence of osmotically selective agents. As different factors could be involved, in the present study we decided to investigate the mechanisms underlying SMPT by individually considering (i) the osmoregulatory mechanisms aimed to upregulate the solute pool, (ii) the possible toxicity of the selective agent in the agar media, and (iii) the selective permeability of the cytoplasmic membrane.

#### Role of the Upregulation of the Solute Pool in SMPT

The synthesis and accumulation of trehalose, or the influx of other osmoprotectants when present in the media, are the result of a cascade of osmoregulatory events triggered in living bacterial cells by osmotic upshocks and intended to maintain their correct turgor pressure (Wood, 2011).

In the present work we explored the osmoregulatory response of WT and mutant cells impaired in trehalose synthesis or in the influx of osmoprotectants, with the objective of determining the involvement of those osmoregulatory mechanisms in SMPT.

##### Upregulation of the solute pool through the accumulation of trehalose

High osmolarity stimulates the transcription of Ots system to synthesize trehalose in media devoid of osmoprotectants, and mutants impaired in *otsA* are osmotically sensitive due to their inability to synthetize trehalose (Lucht and Bremer, 1994). For the present work, we decided to compare the state of untreated or thermally treated WT cells with that of Δ*otsA* cells when plated onto agar with different NaCl concentrations. Also, the proportions of sublethally injured cells were calculated, for each strain and treatment time, by calculating the difference between the survival level in the presence of its MNIC of NaCl and in the absence of NaCl.

**Table 1** shows that, as expected, *E. coli* Δ*otsA* presented a lower NaCl MNIC value (2%) than the WT; the fact that untreated Δ*otsA* cells are unable to grow in agar medium with 3% NaCl is probably due to the absence of the osmoprotectant effect of accumulated trehalose. Furthermore, the reduced osmotolerance of Δ*otsA* cells was also detected in the finding that NaCl concentrations above 8% were capable of inactivating untreated cells (instead of only inhibiting their growth, as observed for the WT cells).

**Table 1:** State of untreated or thermally treated cells after the incubation in M9 agar medium added with each NaCl concentration.

% NaCl in M9 agar	State of WT untreated cells	State of Δ*otsA* untreated cells	State of WT thermally treated cells	State of Δ*otsA* thermally treated cells
0	Viable	Viable	Viable	Viable
1	Viable	Viable	Viable	Viable
2	Viable	Viable	Viable	Inhibited
3	Viable	Inhibited	Inhibited	Inhibited
4	Inhibited	Inhibited	Inhibited	Inhibited
5	Inhibited	Inhibited	Inhibited	Inhibited
6	Inhibited	Inhibited	Non-viable	Non-viable
7	Inhibited	Inhibited	Non-viable	Non-viable
8	Inhibited	Inhibited	Non-viable	Non-viable
9	Inhibited	Non viable	Non-viable	Non-viable
10	Inhibited	Non viable	Non-viable	Non-viable

The application of a prior thermal treatment resulted in the inactivation of otherwise inhibited cells when plated with 6–10% NaCl (**Table 1**). Therefore, we were able to confirm that thermally treated cells of both strains lost their ability to survive in media containing high NaCl concentrations. This observation could be related to the increase in the intracellular accumulation of Na^+^ in cells when plated onto agar with an external osmolality of 2 Os/kg, corresponding to 6% NaCl (Shabala et al., 2009). When considering the proportion of sublethal injury at their respective MNICs, both strains behaved similarly (2,5 ± 0,5 and more than 5 log cycles of sublethal injury after 5 and 20 min of heat treatment respectively, data not shown). The higher osmosensitivity of the mutant lacking the complete trehalose synthesis pathway in comparison with the WT exposes the relevance of trehalose synthesis in SMPT. This finding also agrees with a previously observed reduction in MNIC values of several osmolytes in *E. coli* mutants in the Ots-controlling sigma factor RpoS (Cebrián et al., 2015).

Regarding the specific role of trehalose synthesis or accumulation in the detection of sublethal injury by SMPT, the similar proportions of sublethal injury detected in both strains seem to suggest that, once cells have been thermally damaged, other mechanisms or cellular structures are responsible for their difficulty to outgrow in selective agar media. Further research should be done on the thermosensitivity of Ots as a key factor in the way trehalose and its synthesis pathway are involved in the inhibition and inactivation of sublethally injured cells.

Additionally, an unexpected discovery was made in the results in **Table 1**. Whereas untreated WT cells were inhibited when grown in the presence of concentrations above the MNIC, thermally treated cells remained inhibited when recovered onto agar medium with 3% NaCl, which corresponds to their MNIC and therefore is commonly used to determine the degree of sublethal injury (García et al., 2005). These results contradict, for the first time, the previously accepted hypothesis that sublethally injured cells are inactivated when plated at the MNIC determined for untreated cells (Mackey, 2000): the explanation is that the cells are not being actually inactivated but inhibited in hyperosmotic agar media. For simplicity, throughout the present study we continue to use the term “inactivation” to describe the lack of growth in the recovery medium. On the other hand, this discovery can turn out to be of great relevance, from an applicative point of view, in helping us correctly interpret the lethality of each treatment in combined preservation processes. This is especially true when low water activity is considered as one of the hurdles: since inhibited cells can resume growth under favorable conditions, the error of considering them as inactivated cells would imply that one would underestimate the bacterial content in food and thereby incur in possible health risks for the consumers.

##### Upregulation of the solute pool through the influx of external osmoprotectants

Nutritionally rich agars containing osmoprotectants are commonly used for the detection of sublethal injury in food preservation (Wu, 2008; Wesche et al., 2009). Bacteria take up osmoprotectants from surrounding media via membrane transporters such as ProP or BetT (Haardt et al., 1995; Wood et al., 2001), and their stability could be impaired after thermal treatments and therefore influence the outcome of SMPT. Among osmoprotectants, betaine has been demonstrated to increase growth of *E. coli* cells in hyperosmotic media (Le Rudulier et al., 1984), so *E. coli* mutants lacking the betaine transporter ProP (Δ*proP*) were selected to help determine the role of osmoprotectant transporters in SMPT.

**FIGURE 2:**
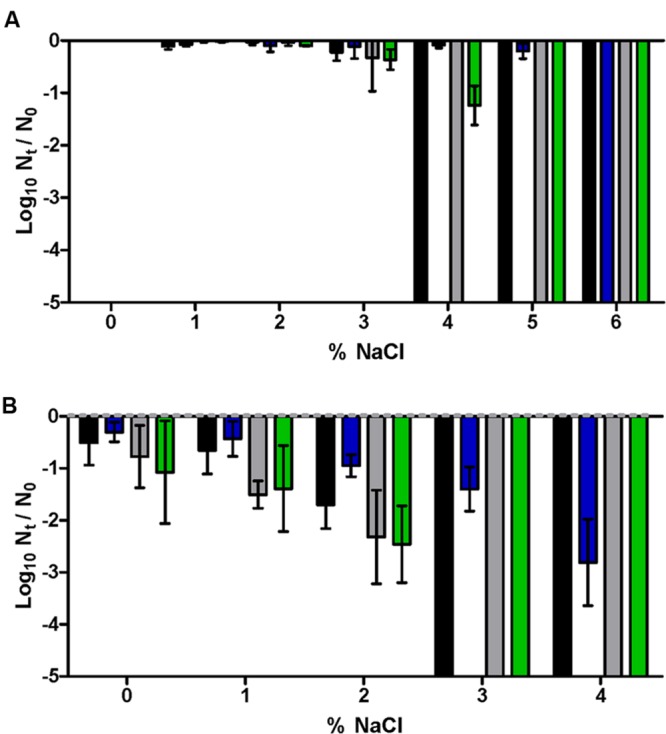
**Log_10_ cycles of survival fractions of untreated **(A)** or treated for 10 min at 55°C in M9 broth (B) of *E. coli* BW25113 WT (black, blue bars) or Δ*proP* (gray, green bars) after incubation in the absence (black, gray bars) or presence (blue, green bars) of betaine 1 mM in the M9 agar of different NaCl concentrations.** Error bars represent standard deviation of the mean from three replicates.

The addition of betaine to the recovery agar medium resulted in an increase in the NaCl MNIC value from 3 to 5% in untreated WT cells, as represented in **Figure 2A** by comparing the black bars (showing inactivation of more than 80% of the initial cell population when plated in the presence of 4% NaCl) with blue bars (showing that inactivation of more than 80% of the initial cell population is only achieved when plated in the presence of 6% NaCl). When WT cells were treated at 55°C for 10 min, a significantly higher proportion of cells were recovered at each % NaCl when recovered in media with betaine, than when betaine was absent (**Figure 2B**; *p* < 0.05). In contrast, **Figure 2B** shows that thermally treated mutants lacking ProP were unable to incorporate betaine: the proportion of growing cells was the same (*p* > 0.05) regardless of the osmoprotectant. Therefore, ProP was still active after thermal treatment (as indicated by the difference between treated WT and Δ*proP* cells when recovered in the presence of betaine), while other transporter(s) possibly responsible for the uptake of betaine in untreated Δ*proP* cells were inactive after the thermal treatment.

**FIGURE 3:**
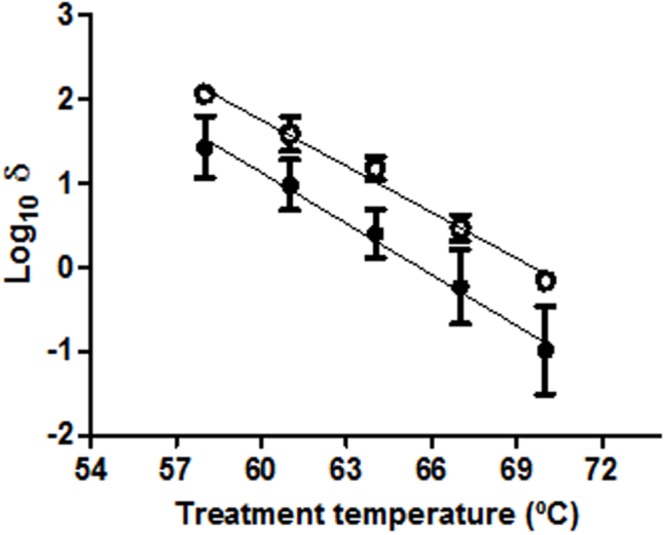
**Log_10_ times for the decimal reduction of *E. coli* BW25113 at different treatment temperatures in M9 broth and recovered in M9 agar added with 3% NaCl in the absence (●) or presence (○) of 1 mM betaine.** Error bars represent standard deviation of the mean from three replicates.

The observation of the remaining activity of ProP after heat prompted us to attempt to ascertain whether more intense thermal treatments could impair ProP and therefore interfere with the detection of sublethal injury. For this purpose, the thermosensitivity of ProP was analyzed by culturing thermally treated WT cells in agar medium containing 3% NaCl, with and without betaine added. Survival curves were modelized so that the time required to inactivate 1 log_10_ cycle of the initial cell population could be compared between the two treatments. According to the results (**Figure 3**), no statistically significant differences were observed between the slopes of the two TDT curves (*p* > 0.05). This implies that the osmoprotectant effect of betaine was maintained throughout the whole range of assayed temperatures (57–70°C), demonstrating the functionality of ProP in the assayed conditions. As a consequence, the possibility that cells might be unable to incorporate osmoprotectants in order to repair their sublethal injury was generally discarded for thermal treatments at temperatures up to 70°C.

On the other hand, the evident influence of added betaine on the osmoregulatory response of *E. coli* and in the results obtained with SMPT using M9 agar medium showed the relevance of the composition of the recovery medium in the interpretation of the sublethally injured fraction via SMPT. Moreover, previous results have demonstrated that the presence of betaine in the recovery medium can compensate for defective phenotypes in their osmoregulatory systems (Cebrián et al., 2015). However, the evaluation of the occurrence of sublethal injury incurred in *E. coli* after inimical treatments via SMPT usually employs complete and nutritionally rich agar media (such as tryptic soy yeast extract agar [TSAYE] or plate count agar) as the non-selective medium (Wuytack et al., 2002; Noriega et al., 2013). In contrast with the controlled and osmoprotectant-free composition of the M9 agar medium, TSAYE contains the osmoprotectant betaine (Dulaney et al., 1968). In our experiments, the presence of betaine in TSAYE was demonstrated by the determination of a MNIC of NaCl at 5% (as in M9 agar medium with betaine), and by the repetition of the treatments applied to obtain **Figure 2** but with TSAYE as the recovery medium: both *E. coli* WT and Δ*proP* behaved in TSAYE similarly as in M9 agar medium with betaine (*p* > 0.05; data not shown).

**FIGURE 4:**
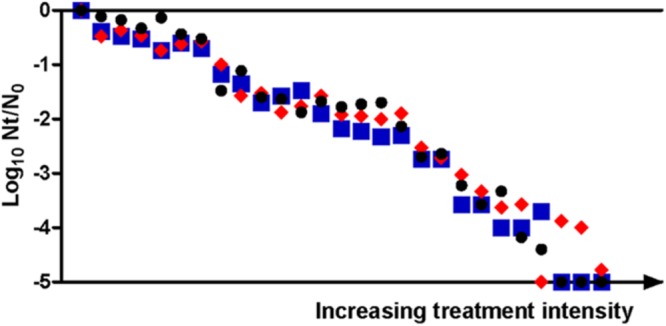
**Log_10_ cycles of survival fractions detected for *E. coli* BW25113 in: M9 minimal agar with its MNIC of NaCl (3%) (●), M9 minimal agar with betaine 1 mM and its MNIC of NaCl (5%) (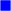) or TSAYE with its MNIC of NaCl (5%) (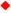).** Thermal treatments of 53–59°C of different durations (0–20 min) were applied in M9 broth to obtain a variety of samples, being each of them depicted in a different column.

In order to control the adequacy of the SMPT using TSAYE as the recovery medium, several thermal treatments of different durations and at different temperatures causing less than 0.5 log_10_ cycles of inactivation in non-selective M9 agar medium were applied to *E. coli* WT cells. The number of log_10_ cycles of inactivation was measured in M9 agar medium, M9 agar medium with betaine and TSAYE, having added their respective MNIC of NaCl (3, 5, and 5%; **Figure 4**). The good correlations obtained between the measurements in the M9 + 3% NaCl agar medium with those in the M9 + 5% NaCl + betaine agar (*R^2^*> 0.95) or with those in the TSAYE + 5% NaCl agar (*R^2^*> 0.92) suggest that, despite the presence of osmoprotectants like betaine, the estimation of the amount of sublethal injury remains constant because of the corresponding increase in the MNIC. Therefore, these results lead to the conclusion that the selection of TSAYE, a recovery agar with osmoprotectants, does not lead to an underestimation of the proportion of sublethally injured cells.

**Figures 3** and **4**, when read together, can lead to a further conclusion. It is easily understandable that only those cells which take up betaine are able to outgrow in agar medium with 5% NaCl, since that osmoprotectant prevents them from being inhibited or inactivated at concentrations above 3% NaCl. However, considering that the applied thermal treatments do not affect ProP (**Figure 3**), the ability to introduce betaine in the cytoplasm is not a limiting factor for heat-treated cells to grow. This would mean that only cells with functional osmoregulatory mechanisms (without considering osmoprotectants) continue to grow after thermal treatments. Given the good correlation between the inactivation detected in treated cells growing in the presence of 5% NaCl with betaine and those growing in the presence of 3% NaCl, it was demonstrated that the latter selective medium is correctly preventing the growth of those cells whose osmoregulation is not completely functional, as previously assumed (Mackey, 2000).

#### Possible Toxicity of Na^+^ in the Cell

Sublethal injury to microbial cell membranes caused by inimical treatments has been linked to the cell’s ability to exclude toxic materials (Gilbert, 1984). Sodium can be considered one of those toxic materials, since *E. coli* cells have to maintain an intracellular Na^+^ concentration lower than the extracellular concentration via the active extrusion systems NhaA and NhaB, regulated by NhaR (Padan and Krulwich, 2000). Moreover, Na^+^ stress is enhanced under conditions in which membrane integrity is compromised, and it has been suggested that in *E. coli* high osmolarity may lead to the induction of specific Na^+^ eﬄux pathways (Padan and Krulwich, 2000).

In order to investigate the possible toxic effect of the presence of Na^+^ in the selective recovery medium, we studied the MNICs of different solutes on untreated cells, as well as their effect on the survival kinetics of thermally treated cells. This way, equivalent osmotic values were achieved in the agar media by incorporation of different concentrations of the ionic osmolytes Na^+^ or K^+^ (as NaCl or KCl), or the non-ionic osmolyte saccharose. In order to facilitate the comparison among osmolytes, the level of inactivation achieved after a very mild thermal treatment (which caused no inactivation in non-selective agar) was obtained in the presence of 25, 50, 75, and 100% MNIC of each osmolyte.

**FIGURE 5:**
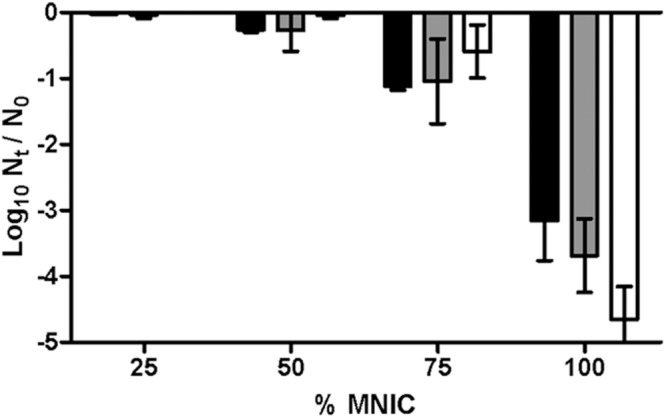
**Log_10_ cycles of survival fractions of *E. coli* BW25113 in M9 broth after 5 min of thermal treatment at 55°C and after incubation in M9 agar added with NaCl (black bars), KCl (gray bars), or saccharose (white bars) at different proportions of their respective MNICs (3.00% NaCl, 3.88% KCl, or 58.53% saccharose).** Error bars represent standard deviation of the mean from three replicates.

The MNIC values of NaCl and KCl were obtained at the same osmolality value (1.02 Os/kg), while the MNIC of saccharose was determined at a greater osmolality value (1.70 Os/kg). In this regard, it has been observed that ionic and non-ionic osmotica trigger different osmoregulatory responses (Shabala et al., 2009). However, this distinction did not seem relevant in SMPT, since similar levels of thermal inactivation (*p* > 0.05) were detected in the presence of the MNIC or lower concentrations of either NaCl, KCl or saccharose (**Figure 5**). Besides, mutants lacking the Na^+^ extrusion systems NhaA, NhaB, or NhaR showed the same MNIC of NaCl than *E. coli* WT cells (3%; data not shown). All these observations suggest that thermal treatments impairing the Na^+^ eﬄux systems could be dismissed as one of the factors intervening in the detection of the sublethal injury under the conditions assayed. This hypothesis agrees with previous observations by Cebrián et al. (2014), who concluded that no specific inhibition mechanisms could be attributed to the ionic osmolytes NaCl or KCl other than the same hyperosmotic stress as imposed by saccharose.

In our attempt to update SMPT, we also noted that not only the MNIC of NaCl and KCl were obtained at the same osmolality value, but also similar *E. coli* survival curves after thermal treatments of 5, 10, or 20 min were obtained for each osmolality value (data not shown). Therefore, although NaCl is commonly used as the selective agent at its MNIC in SMPT (García et al., 2006; Miller et al., 2006; Arroyo et al., 2009), its substitution with KCl could be an alternative possibility.

#### Impairment of Cytoplasmic Membrane Integrity

After the exploration of specific osmoregulatory mechanisms triggered by osmotic upshocks, research into the reason for the inability of sublethally injured cells to outgrow in selective agar medium in SMPT should further explore the physical integrity of the cytoplasmic membrane. Not only is membrane integrity considered to be a key for the maintenance of osmoregulation (Wood et al., 2001), but the inability of cells to overcome the action of the selective agent is considered to reveal structural damage in the cytoplasmic membrane (Wesche et al., 2009). However, little research has been carried out to prove the relationship between the extent of sublethal injury and the physical integrity of the cytoplasmic membrane.

**FIGURE 6:**
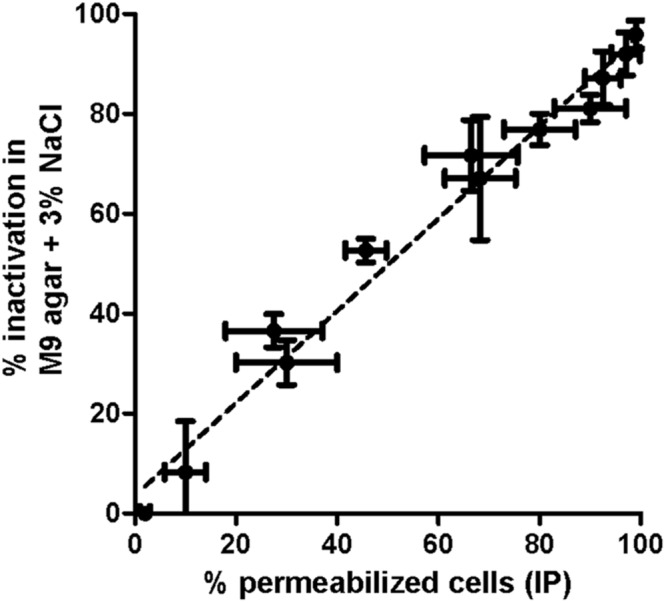
**Correlation and linear regressions between percentage of permeabilization of *E. coli* BW25113 cells after PI staining (measured in fluorescence microscope) and the proportion of inactivated cells (measured by plate count in M9 agar containing 3% NaCl).** Thermal treatments at 55°C of different durations (0–3 min) were applied in M9 broth to obtain a variety of samples. Error bars represent standard deviation of the mean from three replicates.

For the study of membrane integrity, measurement of its degree of permeabilization with the membrane-impermeant dye propidium iodide (PI) has been extensively used to quantify cell damage by penetrating membranes with pores larger than 660 Da (Pagán and Mackey, 2000; García et al., 2005; Kennedy et al., 2011). **Figure 6** shows the correlation between the percentage of permeabilized cells and the level of inactivation measured when thermally treated cells were recovered in agar media with NaCl or KCl at their MNIC. The percentage of permeabilization corresponds to the fraction of cells in which PI had entered through membrane pores during treatment, while the “level of inactivation” factor refers to the percentage of cells – out of the total initial sample population – which were unable to outgrow in the selective agar media (comprising both dead and sublethally injured cells). The total percentage of inactivation – determined by the proportion of cells unable to outgrow in non-selective agar medium – was under 5% even after the longer treatment times of 3 and 5 min (data not shown).

The good correlation between both factors (no significant differences between their slopes, *p* > 0.05), confirms the hypothesis that damage is due to the impairment of membrane permeability (Mackey, 2000; Wesche et al., 2009). **Figure 6** also demonstrates that, independently from the functionality of the osmoregulatory mechanisms, a direct relationship exists between the extent of sublethal injury detected via SMPT and the physical state of the cytoplasmic membrane. It is noteworthy that this good correlation was obtained when PI had been added before the treatment, corresponding to the creation of pores throughout the whole treatment (Pagán and Mackey, 2000). In contrast, the incorporation of PI immediately after each thermal treatment required more than 5 min of thermal treatment in order to lead to the permeabilization of the majority of cells as evidenced by microscopy, and the resulting staining intensity measured by flow cytometry was much lower at any treatment time than when PI was added beforehand (data not shown). This would agree with previous observations which determined that 20-min treatments at 60°C were unable to permeabilize more than 80% of the *E. coli* population via staining with post-treatment PI (Shigapova, 2004; Kennedy et al., 2011). Furthermore, these results agree with the view that PI is a sensitive marker of cell damage, but a poor indicator of cell death (Amor et al., 2002).

### Exploration of the Possible Use of Flow Cytometry as a Complementary Technique to Assess Sublethal Injury

Counting the number of PI-positive cells in terms of percentage only allows for the accurate evaluation of ca. 1 log_10_ cycle of the initial population, as opposed to the 5-log_10_ scale obtained in the previous results by viable plate count. In an attempt to overcome this major limitation, we decided to use flow cytometry as a more appropriate methodology to assess membrane permeabilization through PI uptake. Not only is flow cytometry highly convenient in view of this goal (Amor et al., 2002; Kennedy et al., 2011), but there is also increasing interest in its possible use to complement or even substitute plate count techniques (Nebe-von-Caron et al., 2000; Aronsson et al., 2005).

**FIGURE 7:**
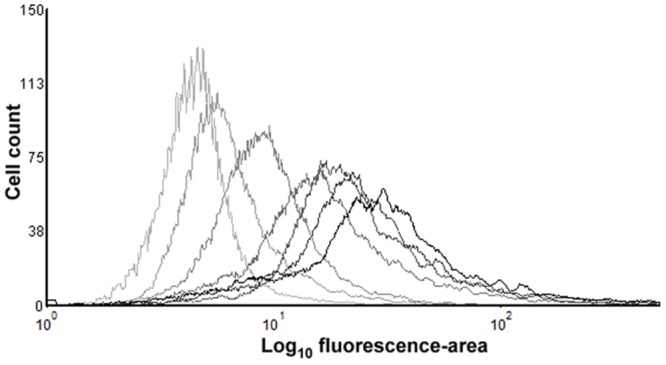
**Fluorescence histogram overlays obtained by flow cytometry measurement of PI-stained *E. coli* BW25113 cells of untreated cells and after thermal treatments at 53°C.** Each overlay represents the frequency distribution of the fluorescence intensity (fluorescence-area values) of a total of 10000 events per sample, corresponding to the untreated sample (lighter overlay), or samples treated for 2, 4, 6, 10, 16, or 20 (darker overlay) min. Results shown from a representative experiment repeated three times with similar results.

**FIGURE 8:**
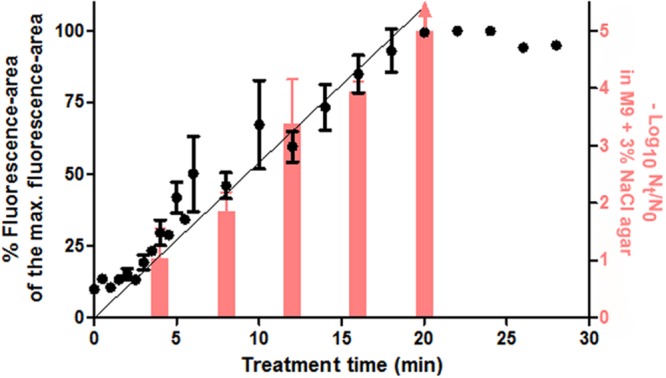
**Median values (●) of the fluorescence-area values obtained from single cell-flow cytometry from PI-stained cells and their linear regression lines, plotted against each duration of the thermal treatment at 53°C.** The graph also shows the log_10_ cycles of inactivation (-log_10_ cycles of the survival fractions) as measured in M9 agar with 3% NaCl (red bars, corresponding to right Y-ax). Error bars represent standard deviation of the mean from three replicates.

In view of this objective, flow cytometer data of PI-stained samples were subjected to statistical analysis and complemented with the measurement of sublethal injury via SMPT. Each overlay in the histogram of **Figure 7** depicts the frequency distribution of the fluorescence intensity’s logarithmic value for a specific treatment time. The position of the overlays alongside the x-axis shows a clear tendency toward increasing fluorescence with longer treatments. Given this observation and the Gaussian aspect of the histogram overlays, we decided to obtain, for each treatment time, a simple parameter characterizing the average fluorescence intensity. For this purpose, the median value of the total of fluorescence-area values of all events was calculated and divided by the maximum average fluorescence achieved by any sample for that assay (**Figure 8**). The inactivation levels detected in M9 agar medium containing 3% NaCl for different treatment times were also plotted (**Figure 8**).

Firstly, we wanted to distinguish between the proportion of stained cells and the total fluorescence value for each sample. In order to calculate the proportion of stained cells, in the data grid obtained through flow cytometry we selected and counted only those events that surpassed the fluorescence threshold. Results showed that the proportion of stained cells increased in parallel with treatment duration for the first 5 min (data not shown). At this point, about 90% of the cells were already stained independently of flourescence intensity (data not shown). Therefore, we confirmed the previously observed correlation (assessed by optical microscopy) between the proportion of permeabilized cells and the proportion of cells unable to grow on the selective medium, since after 4 min of treatment around 85% of cells (nearly 1 log_10_ cycle) had been unable to outgrow in the selective agar medium.

In contrast with the proportion of fluorescent cells, the median value of fluorescence intensities refers to the total fluorescence emitted by the entire bacterial population of each sample, i.e., it assesses the number of PI-stained cells and the fluorescence emitted by each of them. At this point, it should be noted that different staining intensities often occur (Shi et al., 2007), as we have observed from previous experiments using the fluorescence microscope. As can be seen in **Figure 8**, the median values followed a linear evolution throughout treatment time until reaching a maximum value when treatments were 20 min or longer. Therefore, although nearly all the cells had been stained after 5 min of treatment, they were mostly weakly stained, and the fluorescence intensity of the whole population went on increasing throughout treatment at a constant rate. The linearity in the average fluorescence intensity of different samples is a promising concept that has been barely approached. In this regard, Berney et al. (2007) correlated the geometric mean of fluorescence intensity with the amount of nucleic acids, but research could be expanded to different fluorescent probes in order to reveal different grades of a high variety of metabolic processes.

A new methodology for the determination of the occurrence of sublethal injury at a broad detection range (at least 5 log_10_ cycles, depending on sample size) could be developed following the results depicted in **Figure 8**. The meticulous determination of cell plate counts and fluorescence measurements after inimical treatments, as well as calculations of the correlation between both factors, should be performed in order to establish a reference data matrix for further studies. In addition, simultaneous staining with other fluorochromes could provide a better description of the composition of each bacterial sample (Nebe-von-Caron et al., 2000), and therefore help us understand the evolution of treated cells from the viable to dead conditions. From a practical point of view, the rapid detection of the extent of sublethal injury via flow cytometry (and not only the extent of inactivation, as commonly performed) could significantly help in the design of food preservation processes by determining which treatment conditions could be more favorable in the synergistic combination of different hurdles.

### Conclusion on the Evidence of Sublethal Injury through SMPT

According to the results, in SMPT only cells with intact osmoregulatory properties can overcome the osmotic pressure in the selective agar medium (**Figures 3** and **4**). In contrast, those which are considered sublethally injured remain inhibited at the MNIC of the selective agent (**Table 1**). Therefore, cells whose osmoregulatory mechanisms or physical structures become non-functional after thermal treatments are unable to outgrow in osmotically challenging agar media, although they can outgrow in the absence of the selective agent. The identification of such mechanisms or structures, as well as their relationship with the extent of sublethal injury detected, are a key in understanding the performance of SMPT.

In the present study, two of the hypothesized osmoregulatory mechanisms have been discarded as key factors in the performance of SMPT in detecting sublethal injury after heat in *E. coli*: the exclusion of Na^+^ from the cytoplasm and the uptake of osmoprotectants from the agar media. The toxicity of Na^+^ as a cause of sublethal injury had been previously proposed (Gilbert, 1984; Padan and Krulwich, 2000), but we have found evidence neither of Na^+^ toxicity, nor of thermal treatments affecting the Na^+^ extrusion systems. Regarding osmoprotectants, their uptake is absolutely necessary for cells to outgrow in rich media added with NaCl at its MNIC (**Figure 4**). Since the transporter ProP remained active after intense thermal treatments (**Figure 3**), the inability to uptake betaine should not be hypothesized as the reason for the non-survival of sublethally injured cells in selective agar media.

In the absence of osmoprotectants, the main osmoregulatory mechanisms accumulate trehalose. Its absence leads to an increased osmosensitivity and thermosensitivity in untreated and treated cells, and impairs the correct quantification of sublethal injury via SMPT (**Table 1**). However, no direct relationship between the impairment of trehalose synthesis and accumulation systems and the extent of sublethal injury could be established. In contrast, we found a direct relationship between the structural damage of the cell membrane and SMPT via the PI-exclusion technique when PI was added before thermal treatments. In this way, the extent of sublethal injury detected via SMPT could be ascribed to the physical loss of integrity of the cell membrane independently of specific functional osmoregulatory processes. The detection of sublethal injury of *E. coli* after thermal stress has been previously ascribed to the physical loss of integrity of the cell membrane (Mackey, 2000; Ukuku et al., 2008; Wesche et al., 2009). However, to the best of our knowledge, this is the first time that a direct correlation between both factors has been demonstrated, especially at such a high proportion of the bacterial population.

Furthermore, some of the results of the present study can result in the improvement of SMPT. For instance, variations in the composition of the selective media without affecting the outcome of the technique are being proposed: M9 agar + 3% NaCl, M9 agar + 3.88% KCl, M9 agar + betaine + 5% NaCl, and TSAYE + 5% NaCl yielded similar levels of sublethal injury. On the other hand, the possibility of complementing SMPT with flow cytometry to detect bacterial inactivation and injury at a detection range of 5 log_10_ cycles is presented here, since the extent of cell permeabilization (measured simply and rapidly thanks to flow cytometry) was found to be an indicator of the extent of sublethal injury detected with SMPT.

## Conclusion

This work demonstrates, for the first time, that the incorporation in the recovery agar of selective agents which increase its osmotic pressure (such as sodium chloride or potassium chloride) inhibits the growth of *E. coli* cells whose envelopes are physically impaired by mild thermal treatments. Previous hypotheses regarding the implication of two different factors on the performance of SMPT (the toxicity of Na^+^ in the agar and the destruction of transporters of osmoprotectans) were discarded. Moreover, the extent of this physical damage was found to be correlated with the proportion of treated cells unable to grow in selective agar, confirming the adequacy of the SMPT to assess thermal sublethal injury. Further investigation aimed to improve the performance of the SMPT or its combination with flow cytometry could help to maximize its usefulness in food preservation.

## Author Contributions

Conceived and designed the experiments: LE, DG-G, and RP. Performed the experiments: LE. Analyzed the data: LE, DG-G, and RP. Wrote the paper: LE, DG-G, and RP.
